# Overexpression of Abscisic Acid Biosynthesis Gene *OsNCED3* Enhances Survival Rate and Tolerance to Alkaline Stress in Rice Seedlings

**DOI:** 10.3390/plants13121713

**Published:** 2024-06-20

**Authors:** Zhonghui Feng, Yang Xu, Zhiming Xie, Yaqiong Yang, Guanru Lu, Yangyang Jin, Mingming Wang, Miao Liu, Haoyu Yang, Weiqiang Li, Zhengwei Liang

**Affiliations:** 1 College of Life Science, Baicheng Normal University, Baicheng 137000, China; fengzhonghui@bcnu.edu.cn (Z.F.); xiezhiming@bcnu.edu.cn (Z.X.); yangyaqiong2004@163.com (Y.Y.); 2 State Key Laboratory of Black Soils Conservation and Utilization, Northeast Institute of Geography and Agroecology, Chinese Academy of Sciences, Changchun 130102, China; xuyang@iga.ac.cn (Y.X.); luguanru@iga.ac.cn (G.L.); jinyangyang@iga.ac.cn (Y.J.); wangmingming@iga.ac.cn (M.W.); liumiao@iga.ac.cn (M.L.); yanghaoyu@iga.ac.cn (H.Y.); 3 Jilin Da’an Farmland Ecosystem National Observation and Research Station, Da’an 131317, China

**Keywords:** abscisic acid (ABA) content, alkaline tolerance, *OsNCED3*, rice (*Oryza sativa* L.)

## Abstract

Alkaline stress with high pH levels could significantly influence plant growth and survival. The enzyme 9-cis-epoxycarotenoid dioxygenase (NCED) serves as a critical bottleneck in the biosynthesis of abscisic acid (ABA), making it essential for regulating stress tolerance. Here, we show that *OsNCED3*-overexpressing rice lines have increased ABA content by up to 50.90% and improved transcription levels of numerous genes involved in stress responses that significantly enhance seedling survival rates. Overexpression of *OsNCED3* increased the dry weight contents of the total chlorophyll, proline, soluble sugar, starch, and the activities of antioxidant enzymes of rice seedlings, while reducing the contents of O_2_·^−^, H_2_O_2_, and malondialdehyde under hydroponic alkaline stress conditions simulated by 10, 15, and 20 mmol L^−1^ of Na_2_CO_3_. Additionally, the *OsNCED3*-overexpressing rice lines exhibited a notable increase in the expression of *OsNCED3*; ABA response-related genes *OsSalT* and *OsWsi18*; ion homeostasis-related genes *OsAKT1*, *OsHKT1;5*, *OsSOS1*, and *OsNHX5*; and ROS scavenging-related genes *OsCu/Zn-SOD*, *OsFe-SOD*, *OsPOX1*, *OsCATA*, *OsCATB*, and *OsAPX1* in rice seedling leaves. The results of these findings suggest that overexpression of *OsNCED3* upregulates endogenous ABA levels and the expression of stress response genes, which represents an innovative molecular approach for enhancing the alkaline tolerance of rice seedlings.

## 1. Introduction

Based on statistics released by the Food and Agriculture Organization of the United Nations (FAO), approximately 950 million hectares of land across the globe are impacted by soil salinization and alkalization [1]. Saline–alkaline soils can be categorized as either saline or alkaline soils. Saline soil, primarily composed of neutral salts (NaCl or Na_2_SO_4_), typically has a pH level of approximately 7–8. In contrast, alkaline soil, primarily composed of carbonates or bicarbonates (Na_2_CO_3_, NaHCO_3_, or mixtures of Na_2_CO_3_ + NaHCO_3_), usually has a pH value higher than 8.5. Although both saline and alkaline stresses fall under the category of salinization stress, alkaline stress possesses unique characteristics compared with neutral saline stress [2]. The most distinct feature that distinguishes alkaline stress from saline stress is high pH, which causes severe damage to plant growth [3,4,5]. Development of paddy fields for rice (*Oryza sativa* L.) cultivation is an effective method for comprehensive improvement and utilization of soda saline–alkaline soils, which has been jointly discovered through practical experience and scientific exploration [6,7]. Rice production is intricately tied to economic growth. Advancements in rice varieties, cultivation methods, and post-harvest management have led to higher yields and lower costs, making rice more available and affordable for consumers. However, rice is regarded as a crop that is susceptible to the adverse effects of saline–alkaline stress [8,9]. Compared to saline stress, alkaline stress exhibits a more significant inhibitory effect on rice [3], which inhibits plant growth via osmotic stress [10,11], ion toxicity [12,13,14], oxidative damage [14,15,16], and disrupts physiological metabolism [15,16,17]. The seedling stage of rice is the most sensitive to saline–alkaline stress, resulting in a severe decrease in survival rates, leading to seedling death and ultimately affecting crop yield [5,18]. Enhancing the survival rate of rice seedlings is essential in improving the tolerance to alkaline stress.

Abscisic acid (ABA) is a crucial plant hormone that not only regulates plant growth and development but also serves as a stress signal factor mediating plant responses to abiotic stresses [19,20,21,22]. By activating various defense mechanisms, ABA significantly regulates plant responses to adversity, helping plants adapt to potential challenges [23,24]. ABA improves the stability of the cell membrane by elevating the intracellular concentration of Ca^2+^ [25]. Additionally, ABA regulates the expression of stress-responsive genes encoding functionally specific proteins, including enzymes responsible for detoxifying reactive oxygen species (ROS) and enzymes involved in phospholipid-mediated signaling pathways [26]. ABA triggers adaptive responses in plants exposed to saline and alkaline conditions [27]. However, subtle differences might potentially exist in the regulatory mechanisms underlying saline tolerance and alkaline tolerance.

In the majority of higher plants, ABA is produced from carotenoids via the indirect C40 pathway. The catalytic reaction catalyzed by 9-cis-epoxycarotenoid dioxygenase (NCED) serves as a critical rate-limiting step in the biosynthetic pathway of ABA, with the NCED enzyme playing a pivotal role in regulating the overall biosynthetic reaction [28,29,30]. Prior research has demonstrated that the biosynthesis of ABA is modulated by distinct *NCED* genes at various stages of plant tissue growth [31]. In rice, there are five *NCED* genes, namely *OsNCED1*–*OsNCED5*, and different *OsNCED* genes play roles in different biological processes [32,33]. *OsNCED1* and *OsNCED3* are the principal genes regulating ABA biosynthesis in rice, whereas *OsNCED4* does not contribute to this regulatory mechanism [34]. Conversely, *OsNCED5* is involved in the expression of ABA-dependent genes linked to non-biotic stress and senescence, thereby regulating plant development and stress resistance [35]. *OsNCED2* is primarily expressed in seeds and regulates seed germination [33,36]. Furthermore, ectopic overexpression of *OsNCED3* leads to elevated endogenous ABA levels in transgenic lines and enhanced drought tolerance in *Arabidopsis* [37]. Using CRISPR/Cas9 technology, mutant lines of the *OsNCED3* gene, *nced 3-1* and *nced 3-2*, exhibited significantly reduced ABA content and early germination characteristics compared to the wild type (WT) under drought stress [38]. There appears to be a strong correlation between the *NCED* gene family and abiotic stress tolerance in plants. However, reports on the regulation of tolerance to high pH stress in rice by genes associated with ABA biosynthesis remain scarce.

Previous research has revealed that ABA serves as a key factor in improving the tolerance of rice seedlings to saline–alkaline stress, showing broad application prospects in agricultural practices and production [39,40]. However, issues such as high cost and photodecomposition limit the application. Rapid stress-induced increases in endogenous ABA levels and enhancement of ABA signaling are closely related to ABA biosynthesis and metabolism in plants [41]. Therefore, the molecular method of regulating endogenous ABA levels in rice may represent a feasible and effective approach for improving alkaline tolerance. In this study, we overexpressed the critical gene *OsNCED3* involved in the ABA biosynthesis pathway to investigate its function in ABA-mediated alkaline tolerance. As a result, the survival rates and tolerance of rice seedlings towards alkaline stress conditions were significantly improved. This constituted a significant and innovative approach towards enhancing the alkaline tolerance of rice seedlings through the utilization of molecular techniques.

## 2. Results

### 2.1. Overexpression of OsNCED3 Mitigated the Inhibitory Effects of Na_2_CO_3_ Stress Conditions on Plant Growth

**Figure 1:**
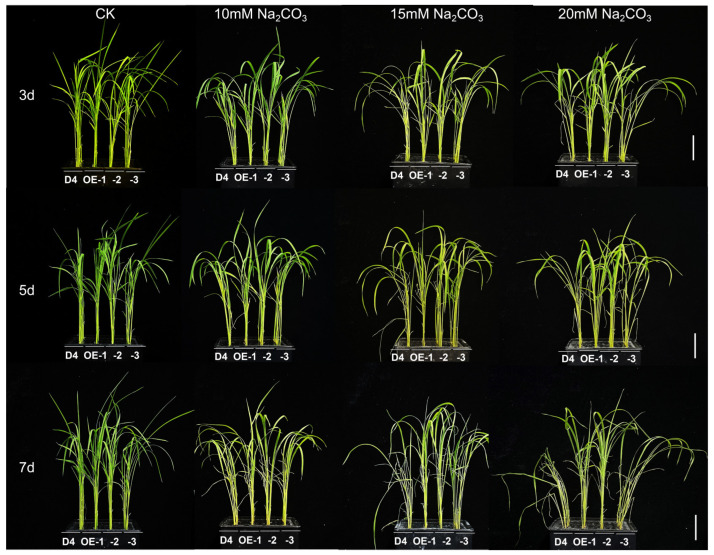
Plant growth of different rice seedling lines under alkaline stress conditions. The growth of D4 (WT) and transgenic line (OE-1, OE-2, and OE-3) rice seedlings at three leaf stages under 10, 15, and 20 mM Na_2_CO_3_ and non-stress conditions (CK, pure nutrient solution) for 3, 5, and 7 d. The scale bar represents 5 cm.

To determine the role of *OsNCED3* in alkaline stress tolerance in rice, we constructed *OsNCED3* transgenic lines (OE-1 to OE-3). With an increase in Na_2_CO_3_ stress concentration and duration, the phenotypic differences between the overexpressing *OsNCED3*-transgenic lines and wild-type (WT) plants became more apparent (Figure 1). The transgenic lines demonstrated significantly superior growth compared to the WT plants when subjected to alkaline stress conditions, with OE-2 showing the best growth phenotype. After 7 d of 20 mM Na_2_CO_3_ stress, the leaves of the WT plants wilted and curled severely, nearly approaching death, while the transgenic lines exhibited significantly less wilting.

### 2.2. Overexpression of OsNCED3 Enhanced the Survival Rates, Dry Weights, and Chlorophyll Contents of Rice Seedlings under Na_2_CO_3_ Stress Conditions

**Figure 2:**
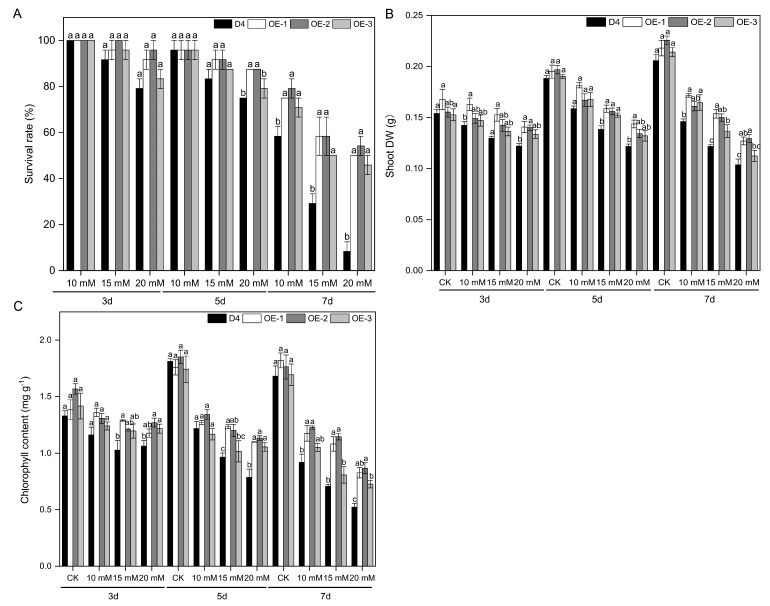
Survival rates, dry weights, and chlorophyll contents of different rice seedling lines under alkaline stress conditions. The survival rates (**A**), dry weights (DWs) (**B**), and chlorophyll contents (**C**) of D4 and transgenic line (OE-1, OE-2, and OE-3) rice seedlings at three leaf stages under 10, 15, and 20 mM Na_2_CO_3_ and non-stress conditions (CK, pure nutrient solution) for 3, 5, and 7 d. Values are means ± SD, *n* = 3. The letters depicted on the column denote statistically significant differences among distinct rice lines subjected to identical treatment conditions based on Duncan’s test (*p* < 0.05).

No statistically significant difference was noted in the survival rates across various lines when the Na_2_CO_3_ stress duration was less than 3 d (*p* < 0.05). However, when the Na_2_CO_3_ stress concentration was higher than 10 mM and the stress duration was longer than 3 days, the survival rates of the transgenic lines were higher than those of the WT plants (Figure 2A). The survival rates of OE-1, OE-2, and OE-3 increased by 5.0-, 5.5, and 4.5 times, respectively, compared to the WT plants under 20 mM Na_2_CO_3_ stress for 7 d, reaching a significant difference (*p* < 0.05).

Alkaline stress also leads to a marked reduction in shoot dry weight and chlorophyll content in rice seedlings. OE-1, OE-2, and OE-3 maintained higher shoot dry weights and chlorophyll content in leaves than the WT under 15 mM and 20 mM Na_2_CO_3_ stress conditions for either 5 or 7 d (*p* < 0.05) (Figure 2B,C).

### 2.3. Overexpression of OsNCED3 Upregulated ABA Contents and the Expression of ABA Response-Related Genes under Na_2_CO_3_ Stress Conditions

**Figure 3:**
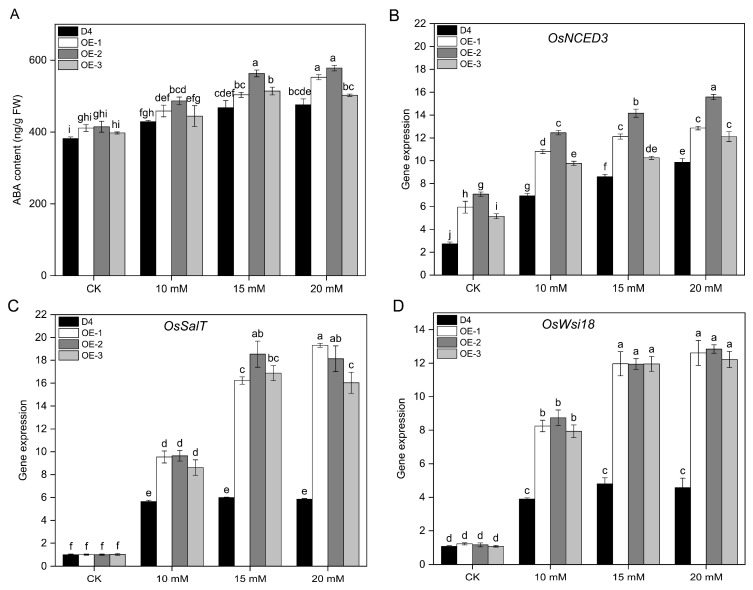
ABA contents and expression levels of *OsNCED3* and ABA-response genes of different rice seedling lines under alkaline conditions. The ABA contents (**A**) of D4 and transgenic line (OE-1, OE-2, and OE-3) rice seedlings at three leaf stages under 10, 15, and 20 mM Na_2_CO_3_ and non-stress conditions (CK, pure nutrient solution) for 3 d. The expression of *OsNCED3* (**B**), *OsSalT* (**C**), and *OsWsi18* (**D**) of D4 and transgenic line (OE-1, OE-2, and OE-3) rice seedlings at three leaf stages under 15 mM Na_2_CO_3_ and non-stress conditions (CK, pure nutrient solution) for 24 h. Values are means ± SD, *n* = 3. The letters depicted on the column denote statistically significant differences among distinct rice lines subjected to different treatment conditions based on Duncan’s test (*p* < 0.05).

Compared to the WT plants, overexpression of *OsNCED3* indicated a significant increase in ABA levels (*p* < 0.05) (Figure 3A). Under 10 mM Na_2_CO_3_ stress, the difference in ABA content between the transgenic lines and the WT remained relatively minimal. Nevertheless, when subjected to higher Na_2_CO_3_ stress concentrations of 15 and 20 mM, transgenic lines demonstrated strikingly elevated ABA content compared to the WT, indicating a significant accumulation effect. Among the transgenic lines, OE-2 exhibited the highest ABA content across different alkaline stress concentrations.

The transgenic lines exhibited significantly elevated expression levels of *OsNCED3* compared to the WT under alkaline stress concentrations (*p* < 0.05) (Figure 3B). The expression of *OsSalT* and *OsWsi18* was significantly upregulated in different rice lines under alkaline stress conditions, with higher upregulation observed in the transgenic lines than the WT plants (*p* < 0.05) (Figure 3C).

### 2.4. Overexpression of OsNCED3 Improved Contents of Osmotic Substances in Rice Seedlings under Na_2_CO_3_ Stress Conditions

**Figure 4:**
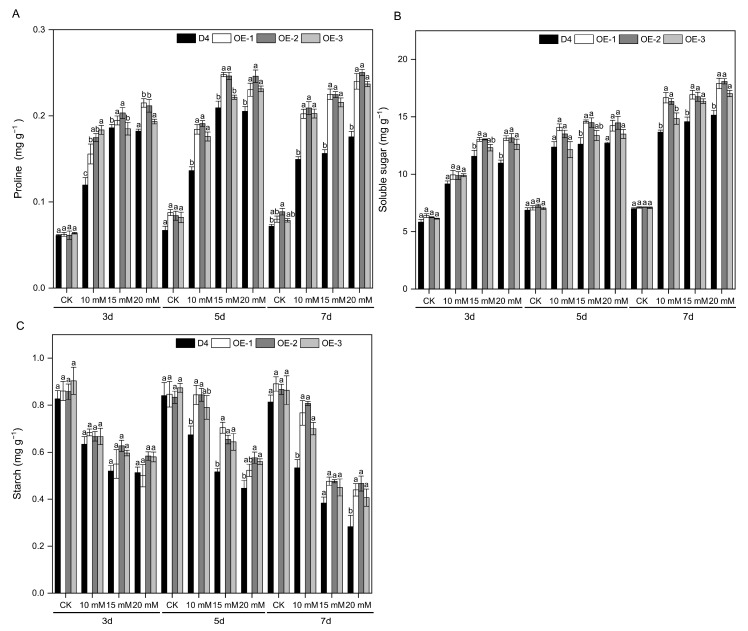
The contents of osmotic substances of different rice seedling lines under alkaline conditions. The contents of proline (**A**), soluble sugar (**B**), and starch (**C**) of D4 and transgenic line (OE-1, OE-2, and OE-3) rice seedlings at three leaf stages under 10, 15, and 20 mM Na_2_CO_3_ and non-stress conditions (CK, pure nutrient solution) for 3, 5, and 7 d. Values are means ± SD, *n* = 3. The letters depicted on the column denote statistically significant differences among distinct rice lines subjected to identical treatment conditions based on Duncan’s test (*p* < 0.05).

Alkaline stress induces accumulation of proline, soluble sugars, and starch in rice seedlings. The transgenic lines OE-1, OE-2, and OE-3 exhibited significantly higher proline, soluble sugar, and starch contents in leaves than WT plants under different concentrations of Na_2_CO_3_ stress (*p* < 0.05) (Figure 4A–C). With an increase in Na_2_CO_3_ stress concentration and duration, differences in contents of proline and soluble sugars became more apparent. In addition, OE-1 and OE-2 accumulated higher contents of osmotic substances than OE-3.

### 2.5. Overexpression of OsNCED3 Regulated Ion Homeostasis of Rice Seedlings under Na_2_CO_3_ Stress Conditions

**Figure 5:**
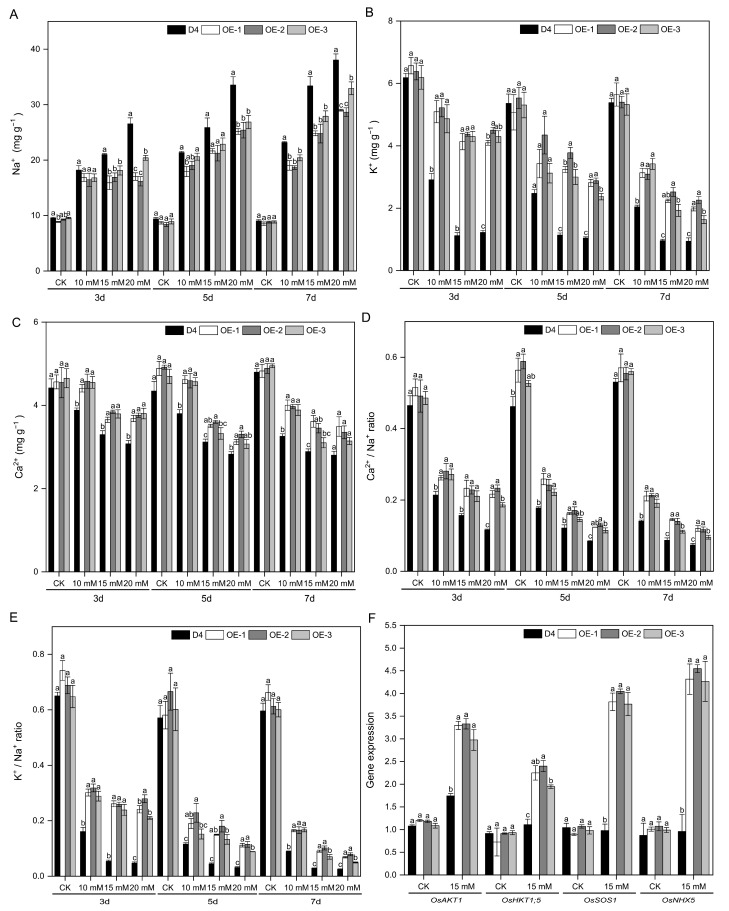
The ion contents and expression levels of ion homeostasis-relative genes of different rice lines under alkaline stress conditions. The contents of Na^+^ (**A**), K^+^ (**B**), Ca^2+^ (**C**), Ca^2+^/Na^+^ ratio (**D**), and K^+^/Na^+^ ratio (**E**) of D4 and transgenic line (OE-1, OE-2, and OE-3) rice seedlings at three leaf stages under 10, 15, and 20 mM Na_2_CO_3_ and non-stress conditions (CK, pure nutrient solution) for 3, 5, and 7 d. The expression of ion homeostasis-relative genes (**F**) of D4 and transgenic line (OE-1, OE-2, and OE-3) rice seedlings at three leaf stages under 15 mM Na_2_CO_3_ and non-stress conditions (CK, pure nutrient solution) for 24 h. Values are means ± SD, *n* = 3. The letters depicted on the column denote statistically significant differences among distinct rice lines subjected to identical treatment conditions based on Duncan’s test (*p* < 0.05).

Alkaline stress resulted in an increased content of Na^+^ of rice seedlings, accompanied by simultaneously decreased contents in both K^+^ and Ca^2+^. Overexpression of the *OsNCED3* gene demonstrated remarkable effectiveness in mitigating the accumulation of Na^+^, enhancing the accumulation of K^+^ and Ca^2+^, elevating both Ca^2+^/Na^+^ and K^+^/Na^+^ ratios in rice leaves subjected to alkaline stress (*p* < 0.05). Specifically, OE-1 and OE-2 exhibited lower Na^+^ content than OE-3 under varying concentrations of Na_2_CO_3_ stress conditions (Figure 5A–D). The Ca^2+^/Na^+^ ratio of OE-1, OE-2, and OE-3 increased by 62.96%, 58.89%, and 29.77%, respectively, compared to WT plants after 7 days of exposure to 20 mM Na_2_CO_3_ stress. Similarly, the K^+^/Na^+^ ratio increased by 1.7-, 2.2, and 1.0 times, respectively.

In comparison with the CK, alkaline stress significantly upregulated the expression of the ion homeostasis-related genes *OsAKT1*, *OsHKT1;5*, *OsSOS1*, and *OsNHX5* in the leaves of different lines (*p* < 0.05), and the expression levels of these genes were higher in the transgenic lines than in the WT plants (Figure 5E,F). Among the different transgenic lines, OE-2 exhibited the highest expression of ion homeostasis-related genes, and the expression levels of these genes were elevated in the transgenic lines compared to the WT plants.

### 2.6. Overexpression of OsNCED3 Reduced Plasma Membrane Damage and ROS Levels under Na_2_CO_3_ Stress Conditions

**Figure 6:**
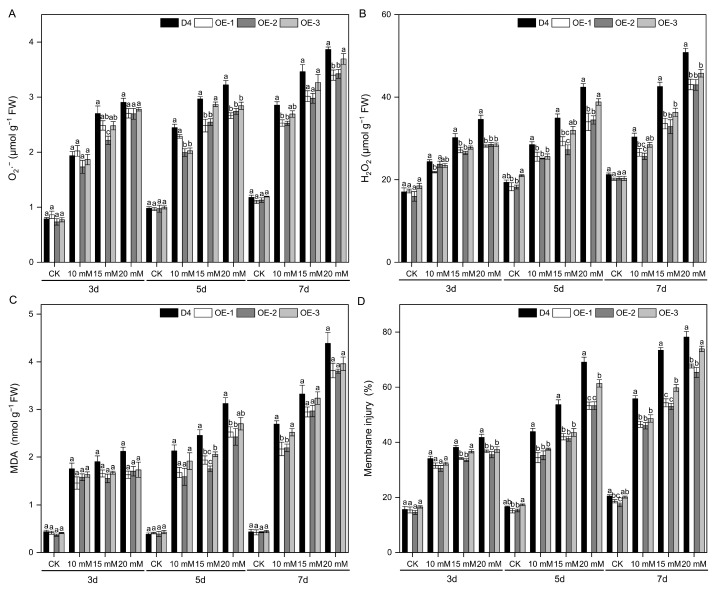
The ROS and MDA levels and membrane injury (MI) of different rice lines under alkaline stress conditions. The contents of O_2_·^−^ (**A**), H_2_O_2_ (**B**), MDA (**C**), and membrane injury (**D**) of D4 and transgenic line (OE-1, OE-2, and OE-3) rice seedlings at three leaf stages under 10, 15, and 20 mM Na_2_CO_3_ and non-stress conditions (CK, pure nutrient solution) for 3, 5, and 7 d. Values are means ± SD, *n* = 3. The letters depicted on the column denote statistically significant differences among distinct rice lines subjected to identical treatment conditions based on Duncan’s test (*p* < 0.05).

There was no substantial variation observed between the WT plants and transgenic lines in terms of plasma membrane injury (MI) or accumulation of reactive oxygen species (ROS) under non-stress conditions. Alkaline stress for 3 days led to increased plasma membrane injury in rice seedling leaves, elevated MDA content, and a sharp increase in the accumulation of O_2_·^−^ and H_2_O_2_. Overexpression of *OsNCED3* resulted in significantly reduced accumulation of O_2_·^−^ and H_2_O_2_, as well as lower MDA content and MI under alkaline stress conditions, among which OE-1 and OE-2 had lower plasma membrane injury and ROS accumulation than OE-3 (*p* < 0.05) (Figure 6A–D).

### 2.7. Overexpression of OsNCED3 Enhanced Antioxidant Defense Capabilities under Na_2_CO_3_ Stress Conditions

**Figure 7:**
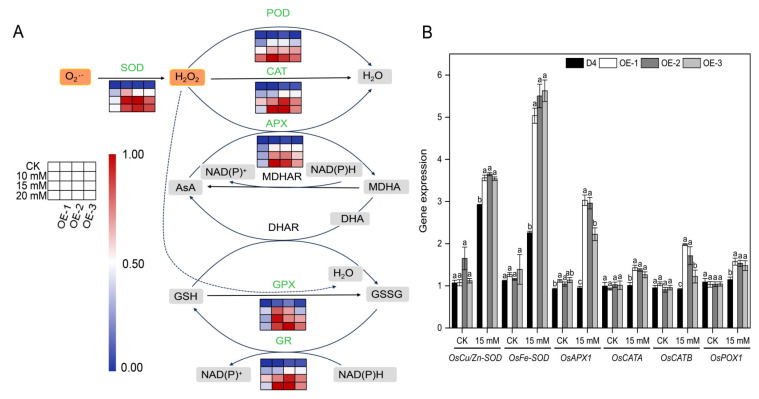
The ROS and MDA levels and membrane injury (MI) of different rice lines under alkaline stress conditions. Activities of antioxidant enzymes (**A**) of D4 and transgenic line (OE-1, OE-2, and OE-3) rice seedlings at three leaf stages under 10, 15, and 20 mM Na_2_CO_3_ and non-stress conditions (CK, pure nutrient solution) for 3 d. The expression of ion homeostasis-relative genes (**B**) of D4 and transgenic line (OE-1, OE-2, and OE-3) rice seedlings at three leaf stages under 15 mM Na_2_CO_3_ and non-stress conditions (CK, pure nutrient solution) for 24 h. Values are means ± SD, *n* = 3. The letters depicted on the column denote statistically significant differences among distinct rice lines subjected to identical treatment conditions based on Duncan’s test (*p* < 0.05).

There was no significant difference in the antioxidant enzyme activity of the leaves between the WT and transgenic lines under non-stress conditions. Alkaline stress for 3 d enhanced the antioxidant enzyme activities of SOD, POD, CAT, APX, GPX, and GR in rice seedling leaves. The antioxidant enzyme activities of the *OsNCED3* overexpression transgenic lines were significantly higher than those of the WT plants under alkaline stress (*p* < 0.05) (Figure 7A), with a more pronounced difference under 20 mM Na_2_CO_3_ stress.

Furthermore, the expression of the ROS scavenging-related genes *OsCu/Zn-SOD*, *OsFe-SOD*, *OsPOX1*, *OsCATA*, *OsCATB*, and *OsAPX1* were significantly higher in the transgenic lines than in the WT plants (*p* < 0.05). Among the different genes, the upregulation of *OsCu/Zn-SOD*, *OsFe-SOD*, and *OsAPX1* was relatively high in the transgenic lines under alkaline stress (Figure 7B).

## 3. Discussion

Approximately 20% of cultivated land and 50% of irrigated land are affected by various degrees of soil salinization [42,43], posing a threat to global food security [44]. Saline–alkaline stress hinders the normal growth, development, and physiological functions of plants [45,46]. Many studies have shown that alkaline stress (dominated by NaHCO_3_ and Na_2_CO_3_) has a much greater impact on plant growth and development than neutral salt stress (dominated by NaCl and Na_2_SO_4_) [3,47]. This is primarily due to the high pH caused by alkaline stress, in addition to osmotic stress and ionic toxicity [48,49].

ABA is a hormone that significantly affects plant growth and metabolism, and it is involved in physiological responses to environmental stresses [22,50,51]. The dynamic balance between endogenous ABA levels in plants is regulated by biosynthesis [52]. Modulating endogenous ABA levels in crops through genetic engineering techniques is one approach to enhance their survival and stress resistance under saline–alkaline conditions. In most higher plants, ABA is synthesized from carotenoids through a C40 indirect pathway, which involves the catalysis of various crucial enzymes. Among these enzymes, 9-cis-epoxycarotenoid dioxygenase (NCED) is a crucial rate-limiting enzyme that plays a pivotal role in the biosynthesis pathway of ABA [53,54]. The rice *OsNCED* gene family comprises a series of genes ranging from *OsNCED1* to *OsNCED5*, all of which play a pivotal role in the modulation of plant resistance mechanisms. Therefore, a comprehensive exploration of the *OsNCED* gene family and its regulatory mechanisms is crucial for enhancing our understanding of alkaline tolerance mechanisms, thereby establishing a theoretical foundation for cultivating rice varieties that exhibit superior resistance. In the present study, we overexpressed the key ABA biosynthesis gene *OsNCED3* in the rice cultivar Dongdao 4 (D4) and obtained the three transgenic rice lines OE-1, OE-2, and OE-3. The results showed that under alkaline stress, transgenic rice seedlings exhibited superior growth trends compared to WT plants (D4), with less wilting and death. Among them, OE-2 exhibited the best phenotypic growth (Figure 1). The survival rates of the transgenic lines overexpressing *OsNCED3* were significantly higher than those of WT plants under alkaline stress (*p* < 0.05) (Figure 2A). Additionally, the overexpression of *OsNCED3* significantly increased the contents of dry weight and chlorophyll under alkaline stress (Figure 3A). Therefore, overexpression of the *OsNCED3* gene can significantly improve the growth of rice seedlings under alkaline stress, alleviate leaf wilting, and enhance seedling survival rates.

The ABA content and *OsNCED3* expression levels in the transgenic lines were significantly higher than those in the WT plants under alkaline stress, and the differences became more apparent with increasing concentrations and durations of alkaline stress (*p* < 0.05) (Figure 3A,B). The OE-2 line had the highest ABA content and *OsNCED3* gene expression levels compared to the other transgenic lines and WT plants under different alkaline stress concentrations. Furthermore, *OsSalT* and *OsWsi18* are key regulatory genes for plant tolerance to stress conditions, respond significantly to both salt stress and ABA treatment in rice, and are considered ABA-responsive genes [25,55]. In the present study, we found that the expression of the ABA-responsive genes *OsSalT* and *OsWsi18* was significantly upregulated in both the wild-type and transgenic lines. However, the transgenic lines exhibited a significantly higher amplitude of upregulation. This suggests that the transgenic lines had enhanced tolerance to alkaline stress, owing to an increase in endogenous ABA levels and activation of the ABA signaling pathway.

Under saline stress, osmolytes play a pivotal role in osmotic adjustment by decreasing the cellular osmotic potential and stabilizing proteins as well as cellular structures [56]. When rice is exposed to saline–alkaline stress, it accumulates osmolytes as osmoprotectants to enhance its resistance [57]. This study revealed that OE-1, OE-2, and OE-3 exhibited significantly higher levels of proline, soluble sugars, and starch in leaves under alkaline stress than the WT plants (*p* < 0.05) (Figure 4). The enhanced ABA biosynthesis in plants potentially modulates the osmotic adjustment mechanism in rice subjected to alkaline stress.

Excessive accumulation of Na^+^ may lead to ion toxicity and damage to plants under saline and alkaline stress. The content of Na^+^ and K^+^ serve as important indicators for assessing plant resistance to saline–alkaline stress [58,59,60]. In the present study, we found that the overexpression of *OsNCED3* significantly reduced the accumulation of Na^+^ in rice leaves under alkaline stress, increased the accumulation of K^+^ and Ca^2+^, and significantly improved the Ca^2+^/Na^+^ and K^+^/Na^+^ ratios (*p* < 0.05) (Figure 5A–E). Maintenance of ion homeostasis is closely related to the regulation of relevant genes. The cell membrane contains various ion channels and transporter proteins that regulate ion influx and efflux, thereby maintaining the ion concentration balance inside and outside the cell. Additionally, intracellular gene transcription regulatory systems are involved in regulating ion balance. For example, the high-affinity potassium (K^+^) transporter 1;5 (*OsHKT1;5*) in rice functions as a Na^+^-selective transporter that mediates Na^+^ efflux through Na^+^ removal from the xylem to maintain the Na^+^/K^+^ balance under salt stress [61]. Wang isolated a salt stress-sensitive mutant, osbag4-1, which exhibited significantly reduced expression of *OsHKT1;5* and K^+^ content, as well as increased Na^+^ content in the shoots [62]. Salt overly sensitive protein 1 (SOS1) and the Na^+^/H^+^ exchanger (NHX) also played crucial roles in the Na^+^ balance [63]. Exposure to high salinity stress triggers calcium signaling, which in turn activates the SOS pathway [64]. The expression of *SsSOS1* is significantly induced by NaCl [65].

The vacuolar Na^+^/H^+^ transporters *OsNHX1*, *OsNHX2*, *OsNHX3*, *OsNHX4*, and *OsNHX5* play an important role in the cytoplasmic accumulation of Na^+^ and K^+^, thereby determining salt tolerance in rice [66]. SOS1, the plasma membrane Na^+^/H^+^ exchanger, plays a key role in the salt tolerance response of rice by promoting Na^+^ expulsion [67]. Members of the high-affinity K+ transporter (HKT) family in rice, including *OsHKT1;1*, *OsHKT1;4*, *OsHKT7*, *OsHKT8*, and *OsHKT1;5*, transport Na^+^ or both Na^+^ and K^+^, assisting in maintaining the Na^+^/K^+^ balance in the cytoplasm and regulating the rice’s response to saline and alkaline stresses [68,69]. In the present study, the overexpression of *OsNCED3* significantly upregulated the expression of the ion homeostasis-related genes *OsAKT1*, *OsHKT1;5*, *OsSOS1*, and *OsNHX5* in rice seedling leaves (Figure 5F). Moreover, exogenous ABA treatment can significantly reduce the Na^+^ content and Na^+^/K^+^ ratios [70], which had similar regulatory effects with the overexpression of *OsNCED3* to change endogenous ABA level in this study on maintaining ion homeostasis in rice seedlings under alkaline stress.

NADPH oxidase (Rboh) is widely recognized as the primary source of H_2_O_2_. Studies have shown that when rice seedlings are subjected to NaCl stress, the *OsRbohA* gene is upregulated in both the shoot and root tissues. However, the tolerance of rice to saline stress is significantly reduced when the *OsRbohA* gene is knocked out [71]. Overexpression lines of *OsMn-SOD* and *OsCu/Zn-SOD* exhibit lower levels of H_2_O_2_ accumulation [72]. The upregulating expression of *OsAPX2*, *OsAPX7*, *OsAPX8*, *OsAPXa*, and *OsAPXb* enhance APX activity, reduce the levels of H_2_O_2_ and malondialdehyde (MDA), mitigate oxidative stress damage, and improve the salt tolerance of rice [73]. Additionally, *OsGR2*, *OsGR3*, and *OsGRX8* increase GSH content and enhance tolerance to various abiotic stresses [74]. In this study, the transgenic lines overexpressing *OsNCED3* exhibited significantly reduced accumulation of O_2_·^−^, H_2_O_2_, and MDA, as well as lower plasma membrane injury in leaves under alkaline stress (*p* < 0.05) (Figure 6). To counter this damage, plants have gradually developed a complex antioxidant defense system during their long-term evolution that includes enzymatic and non-enzymatic ROS scavengers [75]. Enzymatic ROS scavengers include POD, CAT, SOD, APX, GPX, and GR [76]. This study also found that under alkaline stress, the transgenic lines had significantly higher antioxidant enzyme activities than the WT plants and higher expression levels of the ROS scavenging-related genes OsCu/Zn-SOD, *OsFe-SOD*, *OsPOX1*, *OsCATA*, *OsCATB*, and *OsAPX1* (Figure 7). Regulation of the antioxidant enzyme system by overexpressing *OsNCED3* plays a key role in maintaining ROS homeostasis within rice seedling cells, which helps rice adapt to alkaline stress environments. ABA could reduce the accumulation of O_2_·^−^ and H_2_O_2_ in rice leaves under high pH conditions [74,77]. The tolerance of alfalfa to alkaline stress was enhanced by applying ABA to increase antioxidant enzyme activity [35], consistent with the findings of the present study. The overexpression of *OsNCED3* may significantly increase antioxidant enzyme activity and serve as an important defense mechanism against lipid peroxidation and membrane damage caused by alkaline stress.

## 4. Materials and Methods

### 4.1. Plant Material

In our experiment, a locally grown japonica rice cultivar named ‘Dongdao 4’ (D4) was utilized. The D4 was bred through the crossing of ‘Akitakomachi’ and ‘Nongda-10’ in Jilin, China.

### 4.2. DNA Construction and Plant Transformation

The genomic DNA in rice leaves was used as the template for PCR amplification. The full length of *OsNCED3* cDNA (Os03g0645900) sequence was cloned and constructed to the overexpression vector with 35S promoter. The rice cultivar D4 was transformed using the *Agrobacterium* strain EHA105 for rice transformation. For details on the technique used, refer to the protocol described by Toki [78]. Multiple primary transformants, which were designated as T0 plants, underwent self-fertilization to yield T1 generation seeds. T1 transgenic plants exhibiting resistance to hygromycin B were identified by subjecting them to conditions containing 30 μg mL^−1^ of hygromycin B in a medium containing 0.8% agar. These plants showed a segregation ratio of 3:1, favoring resistance to hygromycin B (Hyg-R vs. Hyg-S), which indicated the presence of a single transgene insertion. Subsequently, the selected plants were self-fertilized to produce T2 seeds that were homozygous for the transgene. From this process, three independent homozygous lines of T2 *OsNCED3*-OE were successfully isolated and designated OE-1, OE-2, and OE-3.

### 4.3. Plant Culture Conditions

The rice seeds were first disinfected for 10 min in a 0.1% (*w*/*v*) HgCl_2_ solution, followed by thorough rinsing with distilled water. Then, the seeds were soaked in a beaker filled with distilled water and placed in a constant-temperature incubator at 30 °C for 48 h. After soaking, the seeds were spread at the bottom of a Petri dish and germinated in a constant-temperature incubator set at 28 °C for 24 h. Germinated rice seeds exhibiting uniform germination were planted in 96-well black culture plates and placed in a black plastic culture box 12 mm in length, 87 mm in width, and 114 mm in height. They were grown in distilled water for five days. Afterward, the seedlings were transferred to 1/2 Miyake B nutrient solution [79] and further cultivated in a growth chamber (HPG-400HX; Hadonglian Inc., Beijing, China) under conditions of 25 °C during the light period and 20 °C during the dark period, with a photoperiod of 12 h, until they reached the 3-leaf stage.

### 4.4. Stress Treatments

To simulate alkaline stress treatments with varying degrees, solutions of different concentrations of Na_2_CO_3_ were prepared using analytical grade reagents, specifically 10 mM (pH: 10.61 ± 0.024, EC: 2.33 ± 0.004 mS/cm), 15 mM (pH: 10.82 ± 0.032, EC: 2.80 ± 0.002 mS/cm), and 20 mM (pH: 11.14 ± 0.037, EC: 3.39 ± 0.002 mS/cm). The pH of the solutions was determined using a PHS-25 acidity meter, while the electrical conductivity (EC) was measured utilizing a DDS-12D conductivity meter. To assess the effects of alkaline stress, rice seedlings were treated with 10, 15, or 20 mM Na_2_CO_3_ solutions representing different stress gradients. The control group (CK) was cultivated in nutrient solution without stress. The growth indices and physiological characteristics of rice seedlings were measured on days 3, 5, and 7 under alkaline stress. Each treatment was replicated three times.

### 4.5. Measurement of Survival Rate and Seedling Growth

After six days of alkaline stress, the survival rate of each rice seedling was investigated. Seedlings were considered dead if their leaves were wilted and shriveled and the whole plant was curled up and wilted. Ten seedlings were randomly chosen and deactivated by exposing them to a temperature of 105 °C for an hour, subsequently maintaining them at 70 °C until they reached a constant mass to enable the measurement of their dry weight.

### 4.6. Measurement of Contents of Na^+^, K^+^, and Ca^2+^

The rice samples underwent a drying process by being placed in an oven maintained at 80 °C. Subsequently, 0.1 g of the dried rice leaf samples were accurately measured and transferred into a flask. To facilitate digestion, 4 mL of HNO_3_ and 2 mL of HClO_4_ were added to the flask. Upon completion of the digestion process, the resulting solution was diluted to a total volume of 50 mL. The determination of Na^+^, K^+^, and Ca^2+^ contents in the samples was achieved through use of inductively coupled plasma spectrometry (ICPS-7500, Shimadzu Corporation, Kyoto, Japan).

### 4.7. Measurement of Chlorophyll Content and Endogenous ABA Levels

Chlorophyll content was determined by extraction with a mixed solution of ethanol and acetone [80], with minor modifications. Rice seedling leaves (0.1 g fresh weight) from each treatment were weighed, shredded, and placed in a 15 mL centrifuge tube, and then 10 mL of ethanol–acetone mixed solution (volume ratio 1:1) was added to fully immerse the leaves. When the leaves were completely decolorized and turned white, the absorbance values of the extract at 645 and 663 nm were measured using a spectrophotometer and were denoted as A645 and A663, respectively. ABA content in rice seedlings was determined using an ELISA kit [81].

### 4.8. Measurement of Membrane Injury (MI), Malondialdehyde (MDA) Content, ROS Levels, and Antioxidant Enzyme Activities

The degree of membrane injury (MI) in seedlings was assessed by measuring their relative electrical conductivity, as described by Zhang [82]. The quantification of malondialdehyde (MDA) content was quantified using the thiobarbituric acid (TBA) reaction method [83]. The samples underwent pulverization twice, utilizing a high-throughput tissue crusher operating at 50 Hz for 30 s. Subsequently, 1 mL of a 50 mM phosphate buffer adjusted to a pH of 7.8 was introduced, and the mixture was vigorously vortexed. The homogenate was then centrifuged at 12,000 rpm for 15 min in a temperature-controlled environment of 4 °C. Following centrifugation, 400 µL of the supernatant was combined with 1 mL of 0.5% TBA. The mixture was incubated in a boiling water bath for 20 min, subsequently cooled rapidly, and centrifuged again. The optical density (OD) of the supernatant was assayed at 600 nm, 532 nm, and 450 nm to assess its spectrophotometric properties. The MDA content was calculated using the following formula: 6.45 × (A532 − A600) − 0.56 × A450. Measurement of superoxide anion radical (O_2_·^−^) and hydrogen peroxide (H_2_O_2_) contents and the antioxidant enzyme activities were measured as described previously [16].

### 4.9. Measurement of Proline, Soluble Sugar, and Starch Contents

Dry leaf segments of rice seedlings were measured at 0.1 g, 80% ethanol was added, and the mixture was ground into a homogenous slurry. Afterwards, the slurry underwent a filtering process through filter paper in order to acquire sugar extracts. Subsequently, the content of soluble sugar (SS) was measure. This involved adding 0.2% anthrone to the extract, boiling it for 15 min in a water bath, allowing it to cool to room temperature, and measuring the absorbance value at 620 nm, as per the protocol described by Wei [84]. To determine proline, the ninhydrin colorimetric method was employed with modifications based on the approach outlined by Bates [85]. The starch content was determined by the protocol described by Wang [86].

### 4.10. qRT-PCR

The expression levels of genes related to the ABA response, ion homeostasis, and reactive oxygen species scavenging were detected in each leaf sample stressed for 24 h. The specific primer sequences for all genes are shown in Table S1. Total RNA was extracted (TRIzol reagent, TaKaRa Bio, Tokyo, Japan), followed by reverse transcription (M-MLV reverse transcriptase, Thermo Fisher Scientific, Carlsbad, CA, USA). The qRT-PCR procedure was performed following a previously reported method [16] with *β-actin* (GenBank ID: X15865.1) as an internal standard. The relative expression level was calculated using the 2^−△△CT^ method [87], with each treatment replicated in three biological replicates.

### 4.11. Statistical Analyses

We used IBM SPSS (version 21.0; IBM Corp., Armonk, NY, USA) to conduct comprehensive statistical analyses. Based on the outcomes of the one-way analysis of variance (ANOVA), Duncan’s multiple range test (DMRT) was used to assess significant differences (*p* < 0.05). A graphical representation of the data was meticulously created using Origin 2021.

## 5. Conclusions

Alkaline stress significantly disrupts numerous biochemical and physiological processes, resulting in a reduced survival rate of rice seedlings. Abscisic acid (ABA) plays a positive role in regulating the tolerance of plants to alkaline stress. In summary, the overexpression of the rice ABA biosynthesis gene *OsNCED3* can increase endogenous ABA levels in rice, further activating the ABA signal transduction pathway to enhance osmotic adjustment, ion homeostasis, ROS scavenging, and antioxidant defense. It can also induce the expression of stress response genes, thereby reducing plasma membrane injury and ROS accumulation in seedling leaves, promoting osmotic accumulation, ion balance, and antioxidant defense. These findings provided a highly effective molecular approach to enhancing the alkaline tolerance of rice seedlings. By leveraging modern biotechnology and genetic breeding techniques, we can further explore and harness the potential of the *OsNCED* gene family, offering robust support to agricultural production on saline–alkaline soils. This molecular approach presents a sustainable means of enhancing crop tolerance, thereby revolutionizing agricultural production, even in challenging environments like saline–alkaline paddy fields. In this way, it is possible to help ensure food security and promote sustainable agriculture.

## Data Availability

Data are contained within the article and Supplementary Materials.

## Supplementary Materials

The following supporting information can be downloaded at: https://www.mdpi.com/article/10.3390/plants13121713/s1. Table S1. List of primers used in quantitative real-time PCR analysis.
