# Supplementary material for: Overexpression of Abscisic Acid Biosynthesis Gene OsNCED3 Enhances Survival Rate and Tolerance to Alkaline Stress in Rice Seedlings
Source: Plants (Basel). 2024 Jun 20;13(12):1713. doi: 10.3390/plants13121713 (PMC11207436; doi:10.3390/plants13121713)
Supplement: Supplementary file 1 [file plants-13-01713-s001.zip › plants-3054584-supplementary.pdf]

Table S1. List of primers used in quantitative real-time PCR analysis

Gene name	Gene ID	Forward primer	Reverse primer
OsActin	Os03g0718100	TTCCAGCCTTCCTTCATA	AACGATGTTGCCATATAGAT
OsNCED3	Os03g0645900	GCTCGGTCTCACACACTC	GCTCCCTCTGGTCACTTCC
OsSalT	Os01t0348900	CGAAATAATGTTCCATGGTGTT	TGTACTACGGATCGGTGCAA
OsWsi18	Os01g0705200	TGTGACTCGATCCAGCGTAG	GTTCCTGCTGAGAAGCCATC
OsCu/Zn-SOD	Os08g0561700	TGTGACGGGACTTACTCCTGG	CACCCATTCGTAGTATCGCCA
OsFe-SOD	Os06g0143000	CGACGCCGAGGAATTTCTAG	AGGTGGTGTAAGTGTCTCTCATGC
OsAPX1	Os03g0285700	TCCACCCAGGAAGGGAGG	TTGGTAGCATCAGGAAGACGG
OsPOX1	Os01g0263300	ACAACAACCTCTTCCCGCTA	ATCCAGTCAGAGGCGAGATG
OsCATA	Os02g0115700	CCCCAAGGTCTCCCCTGA	AACGACTCATCACACTGGGAGAG
OsCATB	Os06g0727200	GCTGGTGAGAGATACCGGTCA	TCAACCCACCGCTGGAGA
OsAKT1	Os06g0130900	AGAGATCCTTGATTCACTGCC	TCTACTAACTCCACACTACCAG
OsHKT1;5	Os01g0307500	CCTGCCACCTTACACCACTT	AGCTTCTGCCATATGCTGCT
OsSOS1	Os12g0641100	CTGGGCCTTGCTTTTGGAAT	ATTCCCAGTGTCATGACGGT
OsNHX5	Os09g0286400	TCACTGCCCTTGACAGGAAC	GTCAGGTGGCAACTCATCCA
